# Effect of Poly(Vinyl Alcohol) Concentration and Chain Length on Polymer Nanogel Formation in Aqueous Dispersion Polymerization

**DOI:** 10.3390/molecules28083493

**Published:** 2023-04-15

**Authors:** Yukiya Kitayama, Shunsuke Takigawa, Atsushi Harada

**Affiliations:** 1 Department of Applied Chemistry, Graduate School of Engineering, Osaka Prefecture University, 1-1 Gakuen-cho, Naka-ku, Sakai, Osaka 599-8531, Japan; 2 Department of Applied Chemistry, Graduate School of Engineering, Osaka Metropolitan University, 1-1 Gakuen-cho, Naka-ku, Sakai, Osaka 599-8531, Japan

**Keywords:** nanogel, colloid, polymer, thermoresponsiveness, poly(vinyl alcohol)

## Abstract

Nanotechnology has attracted increasing interest in various research fields for fabricating functional nanomaterials. In this study, we investigated the effect of poly(vinyl alcohol) (PVA) addition on the formation and thermoresponsive properties of poly(*N*-isopropyl acrylamide)-based nanogels in aqueous dispersion polymerizations. During dispersion polymerization, PVA appears to play three roles: (i) it bridges the generated polymer chains during polymerization, (ii) it stabilizes the formed polymer nanogels, and (iii) it regulates the thermoresponsive properties of the polymer nanogels. By regulating the bridging effect of PVA via changing the PVA concentration and chain length, the size of the obtained polymer gel particles was maintained in the nanometer range. Furthermore, we found that the clouding-point temperature increased when using low-molecular weight PVA. We believe that the knowledge gained in this study regarding the effect of PVA concentration and chain length on nanogel formation will aid in the future fabrication of functional polymer nanogels.

## 1. Introduction

Nanomaterials are employed in a wide range of applications, such as biomedical applications [1,2,3,4,5,6,7], catalysts [8], water treatment [9], and dye-adsorbents [10]. Polymer-based nanomaterials are highly attractive because they can be functionalized using a wide range of functional building blocks (monomers) and post-polymerization modifications. Aqueous heterogeneous polymerization systems, such as miniemulsion [11,12,13,14], emulsion [15,16,17,18,19,20], dispersion [21,22,23,24], and precipitation polymerizations [25,26,27,28], are attractive approaches for preparing polymer-based particulate materials from monomer species directly in aqueous media. Among these, dispersion polymerizations with biocompatible stabilizers and precipitation polymerization are attractive for preparing polymeric nanomaterials for bio-related applications. Utilizing these polymerization systems, poly(*N*-isopropyl acrylamide) (PNIPAm)-based microgels/nanogels can be prepared in aqueous media. The PNIPAm-based microgels/nanogels exhibit thermoresponsive properties, owing to the lower critical solution temperature (LCST)-type phase transition property of PNIPAm.

Several researchers have investigated the effect of stabilizers on the particle size and thermoresponsive properties of polymer microgels in the dispersion polymerizations. Among a wide range of stabilizers, poly(vinyl alcohol) (PVA) is a representative biocompatible water-soluble stabilizer in the dispersion polymerizations. In the presence of PVA, the radical chain transfer reaction occurs, and the mid-chain radicals generated on PVA initiate the propagation reaction with monomer species, resulting in covalent grafting of PVA on the polymer gel particles [29]. The grafted PVA chain stabilized polymer particles via steric repulsion. To date, the effect of PVA on the synthesis of poly(NIPAm)-based microgels in dispersion polymerizations has been investigated [30,31]. Yates et al. reported the synthesis of PVA-stabilized PNIPAm-based microgels [30]. Guan and Zhang reported that the addition of PVA significantly affected the thermoresponsive properties of phenylboronic acid-functionalized PNIPAm-based microgels [31]. Furthermore, they investigated the effect of PVA molecular weights on the thermoresponsiveness of the PNIPAm-based microgels.

Nanometer-sized polymer gels (nanogels) with a particle size <100 nm are widely used in drug delivery systems for cancer treatments because of their passive targeting properties derived from enhanced permeability and retention effects [32]. However, PNIPAm-based gels generally attain (sub)micrometer-sized particles in precipitation polymerizations. Nanogels have been successfully synthesized via dispersion polymerization using sodium dodecyl sulfate (SDS) as a surfactant [33]. However, SDS is harmful for biological applications. Recently, we have successfully demonstrated the synthesis of PNIPAm-based nanogels via precipitation polymerization (without surfactants) under suitable conditions [34,35,36,37]. Furthermore, the polymer nanogels can recognize intrinsic dysopsonic proteins (serum albumin) and be employed as novel nanocarriers in drug delivery systems [34,38,39]. These polymer nanogels gain stealth capability in situ in the blood vessel by cloaking themselves with intrinsic serum albumin, resulting in their prolonged circulation in the blood [34]. Furthermore, gold nanoparticle-incorporated polymer nanogels prepared via precipitation polymerization have been successfully used as radiation sensitizers [40]. Therefore, the polymer nanogels prepared by precipitation polymerization have been successfully used as drug delivery carriers.

**Scheme 1:**
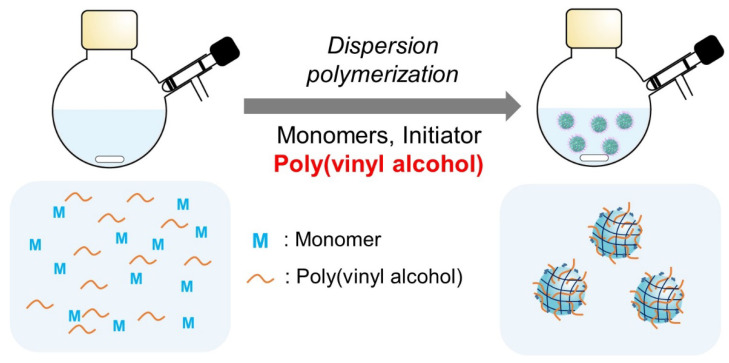
Schematic of preparation of polymer nanogels via aqueous dispersion/precipitation polymerization in the presence of poly(vinyl alcohol).

To the best of our knowledge, no studies examining the effect of PVA addition on polymer nanogel formation have been reported. Herein, we investigated the effect of PVA concentration and chain length on the particle formation and thermoresponsive properties of PNIPAm-based nanogels prepared via aqueous dispersion polymerization (Scheme 1). In a series of experiments, we discovered that PVA plays three roles: (i) it bridges the generated polymer chains during polymerization, (ii) it stabilizes the formed polymer nanogels, and (iii) it regulates the thermoresponsive properties of the polymer nanogels when PVA possessing a low molecular weight (degree of polymerization: 500) is used. We believe that the knowledge gained in this study will aid in the future fabrication of functional polymer nanogels.

## 2. Results and Discussion

### 2.1. Effect of PVA Addition on Nanogel Formation

**Figure 1:**
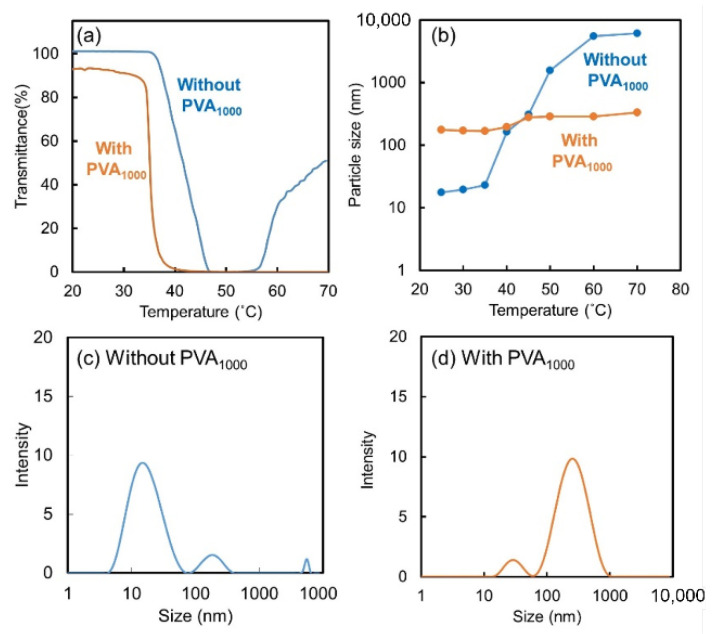
Transmittance (**a**) and average particle size (**b**) of PG_0_ and PG_1000_ dispersions at various temperatures. Particle size distributions of obtained PG_0_ particles (prepared without PVA_1000_, (**c**)) and PG_1000_ particles (prepared with PVA_1000_, (**d**)) at 30 °C. PG_0_ and PG_1000_ particles were prepared via precipitation and dispersion polymerizations with PVA_1000_ (3 mg/mL) at 70 °C for 3 h.

Precipitation polymerization was performed without PVA in 10 mM phosphate buffer (pH 7.4) using *N*-isopropyl acrylamide (NIPAm), *t*-butyl acrylamide (TBAm), 2-methacryloyloxyethyl phosphorylcholine (MPC), and *N*,*N*′-methylenebisacrylamide (MBAA) as monomers. 2,2′-Azobis(2-methylpropionamidine) dihydrochloride (V-50) was selected as the water-soluble initiator. All monomer species and the initiator were dissolved in the solvent prior to polymerization. NIPAm-based polymers are thermoresponsive and exhibit LCST-type phase transitions, owing to the polymer dehydration at high temperatures; thus, the polymers precipitated and assembled as particles to decrease the interfacial free energy during polymerization. The polymers were crosslinked by copolymerizing with MBAA as a crosslinking agent. The conversion of this precipitation polymerization, evaluated using ^1^H-nuclear magnetic resonance (NMR), was approximately 100%, with the protons of the vinyl groups of the monomers almost disappearing (Figure S1). Using dynamic light scattering (DLS), the average particle size of the obtained polymer nanogels (PG_0_) was determined to be approximately 20 nm (Figure 1), which corresponds with that obtained in our previous study [34].

The thermoresponsiveness of PG_0_ was investigated using transmittance measurements. When the temperature reached above the clouding-point temperature of NIPAm-based polymers, their dehydration occurred, resulting in the precipitation of the polymer chains from the aqueous phase. The transmittance of the PG_0_ dispersions steeply decreased above 40 °C, whereas their clouding-point temperature (the temperature at which polymers show 50% transmittance (T)) was estimated to be approximately 43 °C (Figure 1). However, the particle size measured using DLS considerably increased to >1000 nm above the clouding-point temperature, indicating that the polymer nanogels coagulated (Figure 1). The nearly neutral zeta potential of the polymer nanogels (approximately 2.3 mV at 25 °C) indicated that PG_0_ was stabilized via steric repulsion exhibited by hydrated polymer chains grafted onto the polymer nanogels below the clouding-point temperature, rather than electrostatic repulsion between the cationic chain ends of V-50. However, the polymer chains grafted onto the nanogels do not exhibit steric repulsion above the clouding-point temperature due to dehydration, causing particle coagulation.

**Figure 2:**
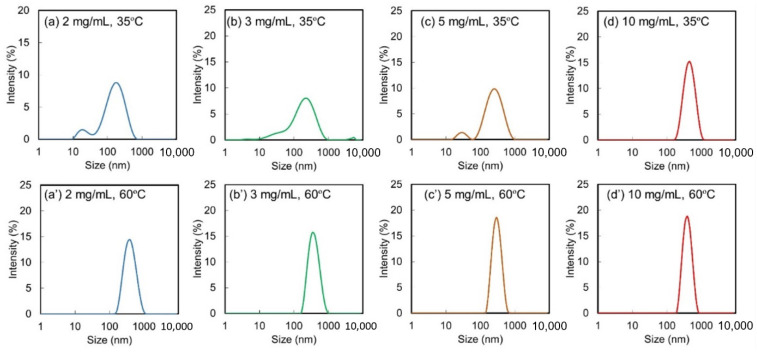
Particle size distributions of PG_1000_ particles at 35 °C (**a**–**d**) and 60 °C (**a**′–**d**′). PVA_1000_ concentrations: 2 (**a**,**a′**), 3 (**b**,**b′**), 5 (**c**,**c′**), and 10 (**d**,**d′**) mg/mL. PG_1000_ particles were prepared via dispersion polymerization with different PVA_1000_ concentrations (2, 3, 5, and 10 mg/mL) at 70 °C for 3 h.

To investigate the effect of PVA on the particle formation, PVA_1000_ (polymerization degree: 1000; average saponification degree: 88%, 3 mg/mL) was added to the polymerization system. PVA is widely used in biomedical applications as a nanocarrier material or bioadhesive because of its low toxicity [41,42]. Therefore, the PNIPAm-based nanogels were prepared in the presence of PVA_1000_ (PG_1000_). The conversion reached approximately 100% even in the presence of PVA, as evaluated using ^1^H-NMR spectroscopy (Figure S2). This is because the radical concentration in the polymerization system does not significantly change when the radical chain transfer to PVA occurs. The clouding-point temperature of the obtained PG_1000_ (approximately 40 °C) estimated from the transmittance measurements was similar to that of the PG_0_ (approximately 43 °C) (Figure 1), indicating that the additional amount of PVA_1000_ had no significant effect on the phase transition temperature of the gel particles. The transmittance of the gel particle dispersion was reversibly changed by heating and cooling (Figure S3). However, the particle sizes of PG_0_ and PG_1000_ significantly differ. Below the clouding-point temperature, PG_1000_ had a larger average particle size (approximately 125 nm) than PG_0_ (approximately 20 nm). Above the clouding-point temperature, the average particle size of PG_1000_ remained constant at approximately 350 nm (Figure 1). The particle size distribution of PG_1000_ below the clouding-point temperature indicates the bimodal distribution of nanometer-sized and submicrometer-sized particles. However, the particle size distribution was unimodal above the clouding-point temperature (at 60 °C), and the nanometer-sized particles disappeared from the distribution (Figure 2). These results indicate that the nanometer-sized gel particles coagulated with the submicrometer-sized gel particles above the clouding-point temperature. To investigate whether particle coagulation occurred above clouding-point temperatures, particle size measurements were performed after adding a cationic surfactant [cetyltrimethylammonium bromide (CTAB)] because cationic surfactants suppress the coagulation of destabilized particles above the clouding-point temperatures. Notably, above the clouding-point temperature, the particle size of PG_1000_ did not significantly increase in the presence of a cationic surfactant (Figure S4). These results indicate that the increase in the particle size of PG_1000_ above the clouding-point temperatures was caused by the coagulation of the destabilized polymer nanogels.

**Scheme 2:**
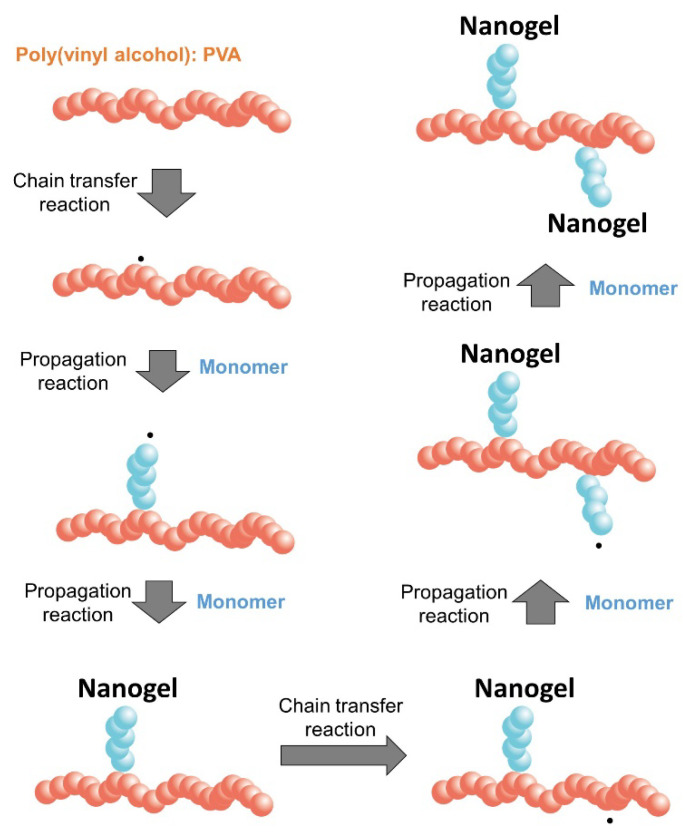
Schematic of the bridging effect of PVA between different polymer chains in nanogels.

Based on these results, PVA appears to play two roles in the particle formation/stabilization: as a stabilizer (primary role) and as a bridging ligand (secondary role). During polymerization, mid-chain radicals are generated on PVA via a chain transfer reaction, which initiates the monomer addition reaction. The radical species then undergo a termination reaction with another radical species, yielding dead polymers. During dispersion polymerization, the propagating/dead polymer chains precipitate from the aqueous phase. This series of reactions (chain transfer, propagation, and termination) graft PVA chains on the polymer nanogels, leading to particle stabilization via steric hindrance (primary role of PVA). However, the series of reactions can occur multiple times on the same PVA, inducing bridging between two or more polymer nanogels with a single PVA chain (Scheme 2). The bridging phenomenon (secondary role of PVA) may lead to the formation of submicrometer-sized polymer nanogels.

### 2.2. Effect of PVA_1000_ Concentration

**Figure 3:**
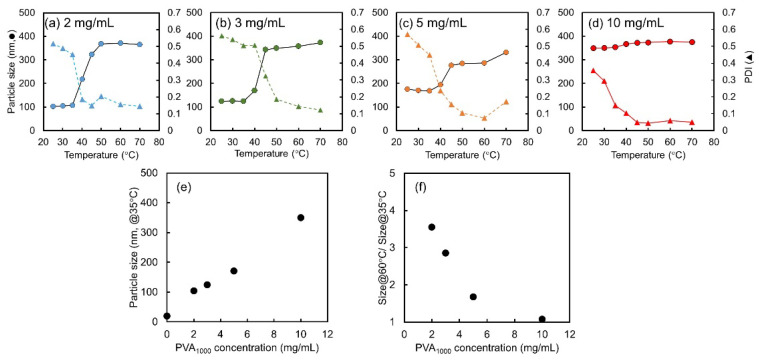
Average particle sizes (circles) and polydispersity index (PDI, triangles) (**a**–**d**) of PG_1000_ particles at various temperatures, where PG_1000_ particles were prepared with 2 (**a**), 3 (**b**), 5 (**c**), and 10 mg/mL (**d**) of PVA_1000_ concentrations. Average particle size (at 30 °C) (**e**) and size ratio (at 60 °C vs. at 30 °C) (**f**) of PG_1000_ at various PVA concentrations.

**Scheme 3:**
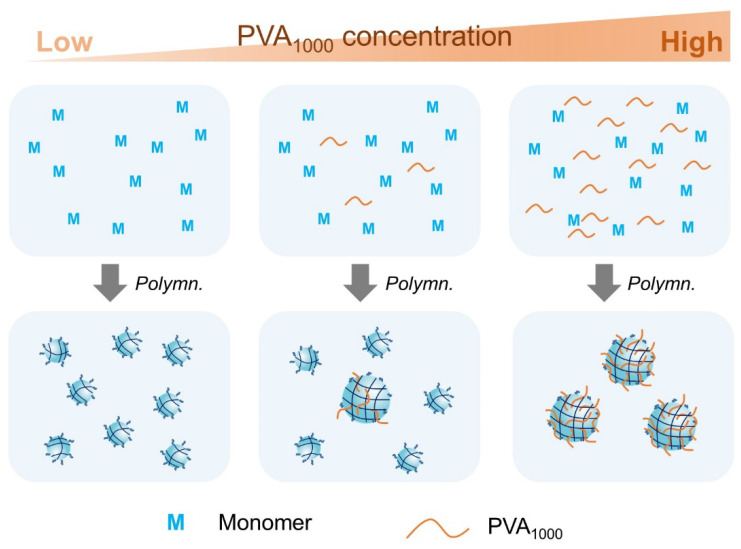
Schematic of the effect of the PVA_1000_ concentration on nanogel formation.

Based on the above findings, we can speculate that the submicrometer-sized gel particles were formed via the PVA_1000_-induced bridging of multiple nanogels, whereas the nanometer-sized gel particles might have formed without the bridging effect. To verify this hypothesis, we investigated the effect of the PVA_1000_ concentration on particle formation and stabilization. When the PVA_1000_ concentration was increased from 2 to 10 mg/mL (at 35 °C, below the clouding-point temperature), the average size of the gel particles gradually increased (Figure 3), and the peak corresponding to the nanometer-sized gel particles disappeared when 10 mg/mL of PVA_1000_ was used (Figure 2). These results indicate that the submicrometer-sized gel particles were formed because of the bridging effect of PVA_1000_. Notably, the clouding-point temperatures determined using the transmittance measurements were similar for all PVA_1000_ concentrations, indicating that the PVA_1000_ concentration did not affect the phase transition temperature of PG_1000_ during polymerization (Figure S5). Furthermore, the particle size distributions obtained all PVA_1000_ concentrations were unimodal above the clouding-point temperature (at 60 °C), indicating that the submicrometer-sized polymer gel particles were colloidally stable even above the clouding-point temperature (Figure 2). These results clearly indicate that the addition of PVA_1000_ led to both the stabilization and bridging of polymer gel particles and/or polymer chains during polymerization (Scheme 3). Thus, the polymer gel particles may have crosslinked by MBAA and PVA. Furthermore, the grafting density of the polymer gel particles may increase with increasing PVA concentration because PVA can act as a bridging agent (crosslinker) between different nanogel particles and/or polymer chains. However, a detailed investigation is necessary to evaluate the crosslinking density of the polymer gel particles using light or X-ray scattering.

### 2.3. Effect of PVA Chain Length

To obtain stable polymer nanogels, we regulated the bridging and stabilization effects of PVA on the obtained polymer nanogels by changing the PVA chain length. We investigated the effect of the PVA chain length on the particle size and colloidal stability using three different PVAs with varying degrees of polymerization (500: PVA_500_, 1000: PVA_1000_, and 3500: PVA_3500_). When PVA with a longer chain length was used, the PVA-induced bridging effect of different polymer chains/nanogels was increased, and the large gel particles with high colloidal stability were obtained. However, the bridging effect decreased when PVA with a shorter chain length was used, and small gel particles were obtained owing to the suppression of the bridging effect of PVA.

**Figure 4:**
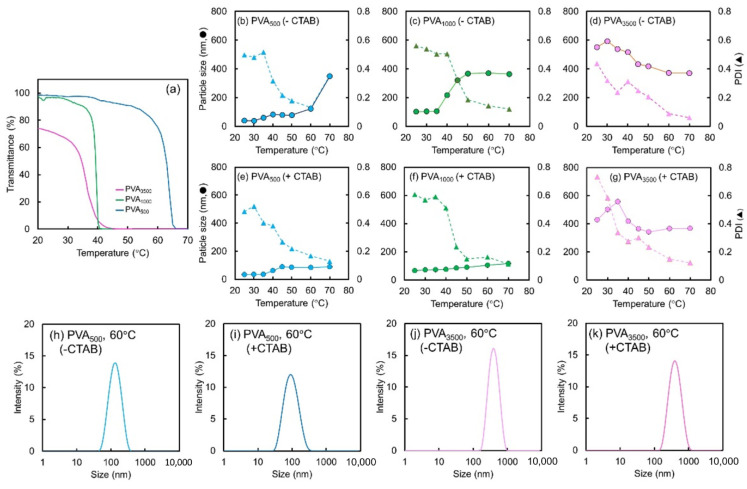
Transmittance of PG_500_, PG_1000_, and PG_3500_ particle dispersions prepared via dispersion polymerization (**a**). Average particle sizes (circles) and PDI (triangles) of PG_500_ (**b**,**e**), PG_1000_ (**c**,**f**), and PG_3500_ (**d**,**g**) particles at various temperatures without (**b**–**d**) or with (**e**–**g**) CTAB. Particle size distributions of PG_500_ (**h**,**i**) and PG_3500_ (**j**,**k**) particles at 60 °C without (**h**,**j**) and with (**i**,**k**) CTAB. Polymer gel particles were prepared via dispersion polymerization with PVA polymer chains of different lengths (polymerization degrees: 500, 1000, and 3500) at 70 °C for 3 h.

Figure 4 shows the transmittance of the obtained polymer gel particle dispersions prepared using three different types of PVA. When the polymerization degree of PVA was increased to 3500 (PVA_3500_), the clouding-point temperature of the obtained polymer gel particles (PG_3500_) was not significantly shifted to a lower temperature compared to that of PG_1000_. Furthermore, the transmittance below the clouding-point temperature was slightly low (~75%), implying the formation of larger polymer gel particles. In fact, submicrometer-sized gel particles were obtained using PVA_3500_ (PG_3500_) below the clouding-point temperature. These results support our hypothesis that PVA assists the bridging of different polymer chains/nanogels during dispersion polymerization. Importantly, the thermoresponsiveness of PG_3500_ was different from that of PG_1000_. The particle size of PG_1000_ significantly increased above the LCST, whereas that of PG_3500_ decreased (Figure 4). This phenomenon exhibited by PG_3500_ is observed for submicrometer-sized gel particles with LCST-type thermoresponsive properties [43]. Furthermore, PG_3500_ particles showed a similar decreasing trend even in the presence of CTAB. This implies that PG_3500_ did not coagulate, but rather shrank because of dehydration above the clouding-point temperatures.

**Figure 5:**
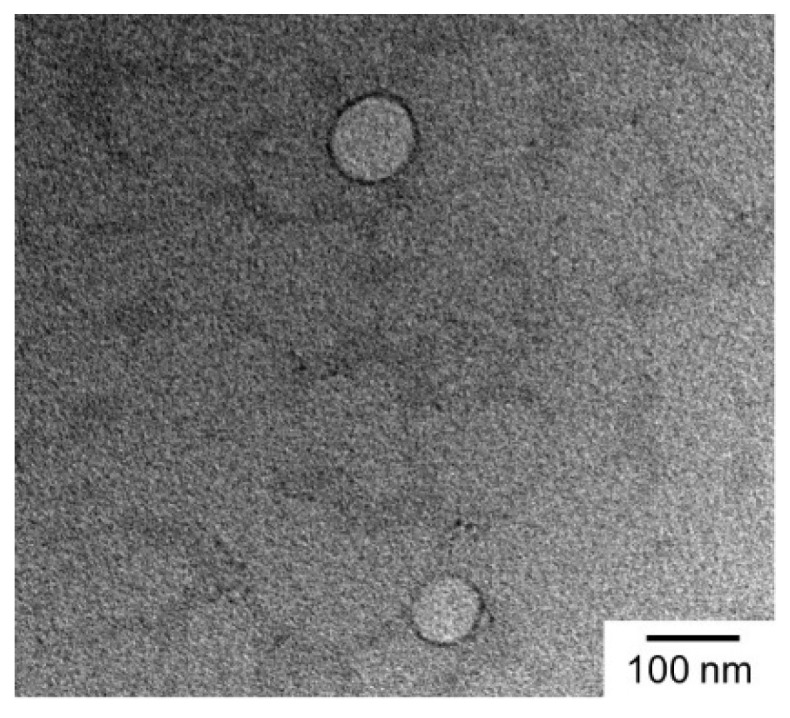
Transmission electron microscopy image of PG_500_ prepared via dispersion polymerization with PVA_500_.

The thermoresponsive properties and colloidal stability of the polymer gel particles prepared using PVA_500_ (PG_500_) were different from those of PG_1000_ and PG_3500_. The particle size of PG_500_ was less than 100 nm at temperatures lower than the clouding-point temperature (Figure 4). Furthermore, the nanometer-sized polymer nanogels were clearly visible in transmittance electron microscopy images when the PNIPAm-based nanogels were prepared with PVA_500_ (Figure 5). The nanometer size of PG_500_ particles was maintained up to 60 °C, while the particles coagulated above 65 °C (Figure 4). The clouding-point temperature estimated from the transmittance measurements of PG_500_ was approximately 63 °C, which was higher than those of PG_1000_ and PG_3500_. This may be attributed to the insertion of PVA_500_ into the polymer main chains. Previous studies have revealed that the copolymerization of a hydrophilic monomer with PNIPAm shifts the LCST to a higher temperature [44]. Furthermore, a negligible increase in the particle size of PG_500_ was observed above 60 °C when CTAB was added during DLS measurements. These results indicate that the increase in particle size of PG_500_ (without CTAB) above 60 °C was caused by coagulation. The colloidal stability of PG_500_ at high temperatures (up to 60 °C) was induced by the steric repulsion of grafted PVA_500_. These results suggest that the bridging effect between the different polymer nanogels weakened with decreasing polymerization degree of PVA, whereas PVA-induced colloidal stability was afforded to the polymer nanogels. Therefore, we can conclude that the different degrees of polymerization of PVA affected the formation of polymer gel particles during dispersion polymerization by controlling the bridging effect between different polymer chains/nanogels. Furthermore, to investigate whether PVA was covalently bonded in PNIPAm nanogels, we prepared the PNIPAm nanogels with fluorescein-labeled PVA. The fluorescence spectrum of the fluorescein-labeled PVA-containing polymer gels as a dispersed state was obtained (Figure S6). The spectra clearly indicate that the PVA was covalently bonded in PNIPAm nanogels.

## 3. Conclusions

In this study, we investigated the effect of PVA addition on the polymer nanogel formation during dispersion polymerization. Our results indicate that PVA induces three effects on the polymer nanogels: (i) bridging between different polymer chains formed during polymerization, (ii) stabilization of the polymer nanogels, and (iii) regulation of thermoresponsiveness. In the presence of PVA with a higher degree of polymerization (PVA_3500_), microgel particles were formed during dispersion polymerizations, owing to the enhanced bridging effect. On the contrary, the nanogels that are stable over a wide temperature range (under 60 °C) were obtained using PVA_500_. The nanotechnology developed herein may help in further advancing research on polymer nanogels in fields such as biomedicine, wherein the fabrication of nanocarriers for targeted drug delivery and other similar functions may be possible.

## 4. Materials and Methods

### 4.1. Materials

NIPAm, MBAA, and potassium dihydrogen phosphate were purchased from Nacalai Tesque (Kyoto, Japan). TBAM, PVA with different degrees of polymerization and saponification, disodium hydrogenphosphate, V-50, and VA-044 were purchased from Wako Pure Chemical Co., Ltd. (Osaka, Japan). MPC, acryloxyethyl thiocarbamoyl rhodamine B, and CTAB were purchased from Sigma–Aldrich (St. Louis, MO, USA). Deionized (DI) water was obtained using a Millipore Milli-Q purification system. FAm was prepared using a previously reported procedure [9].

### 4.2. Apparatus

UV-visible (UV-Vis) spectral measurements were conducted using a V-560 spectrophotometer (Jasco Ltd., Tokyo, Japan). ^1^H NMR spectra were measured using a 400-MHz Fourier transform (FT)-NMR apparatus (JNM-ECX400 FT-NMR system, JEOL Ltd., Tokyo, Japan). The particle size distribution and the zeta potential of the obtained particles were measured using ZETASIZER NANO-ZS (Malvern, UK).

### 4.3. Precipitation/Dispersion Polymerizations

NIPAm (407 mg, 3.6 mmol), TBAm (7.6 mg, 60 μmol), MPC (30 mg, 0.1 mmol), FAm (4 mg, 11 μmol), MBAA (30.8 mg. 0.2 mmol), and V-50 (217 mg, 0.8 mmol) were mixed with 10 mM phosphate buffer (pH 7.4, 100 mL) in a Schlenk flask. After N_2_/vacuum cycles, polymerization was performed at 70 °C for 3 h. To investigate the effect of the PVA concentration, various amounts (final concentration: 0–10 mg/mL) of PVA (polymerization degree: 1000, saponification degree: 88%) were added to the solution. To investigate the effect of the polymerization degree of PVA, the same concentrations (final concentration: 3 mg/mL) of PVA with different polymerization degrees (500, 1000, and 3500) and 88% saponification were added to the polymerization system.

### 4.4. Transmittance Measurements

As-prepared particle dispersions (3 mL) were poured into the UV-Vis cell, and the transmittance measurement (*λ* = 600 nm) was conducted. The temperature was increased at a constant rate (0.5 °C/min). The reversibility of the thermoresponsiveness of the obtained particles was evaluated by alternately increasing and decreasing the temperature at a constant rate (0.5 °C/min).

### 4.5. Particle Size Distributions

The particle size distributions of the as-prepared particle dispersions (3 mL) were measured using DLS at different temperatures (25, 30, 35, 40, 45, 50, 60, and 70 °C). The measurements were performed sequentially at 600 s intervals. To investigate the effect of surfactants on particle aggregation, CTAB (~1 mg/mL) was added to the particle dispersion.

## Data Availability

The data presented in this study are available in the article.

## Supplementary Materials

The following supporting information can be downloaded at: https://www.mdpi.com/article/10.3390/molecules28083493/s1. Figure S1: The ^1^H-NMR spectra of precipitation polymerization, Figure S2: The ^1^H-NMR spectra of dispersion polymerization with PVA, Figure S3: reversibility of the transmittance changes of polymer nanogel dispersion, Figure S4: particle size change of nanogels in the presence of cationic surfactants, Figure S5: and clouding-point temperatures of PG_1000_ prepared with different PVA_1000_ concentrations, Figure S6: Dispersion polymerization with fluorescein-labeled PVA_500_.

## Sample Availability Statement

Samples of the compounds are not available from the authors.

## Abbreviations

CTAB: cetyltrimethylammonium bromide; DLS, dynamic light scattering; FAm, fluorescein acrylamide; LCST, lower critical solution temperature; MBAA, *N*,*N*′-methylenebisacrylamide; MPC, 2-methacryloyloxyethyl phosphorylcholine; NIPAm, *N*-isopropyl acrylamide; PVA, poly(vinyl alcohol); TBAm, *t*-butyl acrylamide; V-50, 2,2′-azobis(2-methylpropionamidine) dihydrochloride; NMR, nuclear magnetic resonance.
