# Supplementary material for: Effect of Poly(Vinyl Alcohol) Concentration and Chain Length on Polymer Nanogel Formation in Aqueous Dispersion Polymerization
Source: Molecules. 2023 Apr 15;28(8):3493. doi: 10.3390/molecules28083493 (PMC10143144; doi:10.3390/molecules28083493)
Supplement: Supplementary file 1 [file molecules-28-03493-s001.zip › molecules-2310383-supplementary.pdf]

Supporting Information

Effect of Poly(vinyl Alcohol) Concentration and Chain Length on Polymer Nanogel Formation in Aqueous Dispersion Polymerization

Yukiya Kitayama[1,2], Shunsuke Takigawa[1], and Atsushi Harada[1,2]**

1 Department of Applied Chemistry, Graduate School of Engineering, Osaka Prefecture University, 1-1, Gakuen-cho, Naka-ku, Sakai, Osaka 599-8531, Japan

2 Department of Applied Chemistry, Graduate School of Engineering, Osaka Metropolitan University, 1-1, Gakuen-cho, Naka-ku, Sakai, Osaka 599-8531, Japan

* Correspondence: kitayama@omu.ac.jp (Y.K.); atsushi_harada@omu.ac.jp (A.H.)

1. ^{1}H-NMR in Precipitation Polymerization without PVA 2
2. ^{1}H-NMR in Dispersion Polymerization with PVA_{1000} 3
3. Transmittance Measurements of PG_{1000} 4
4. Particle Size of PG_{1000} in the Presence of CTAB 5
5. Clouding-Point Temperature of PG_{1000} Prepared with Different PVA_{1000} Concentrations 6
6. Dispersion Polymerization with Fluorescein-labeled PVA_{500} 7

Contents

1. ^{1}H-NMR in Precipitation Polymerization without PVA

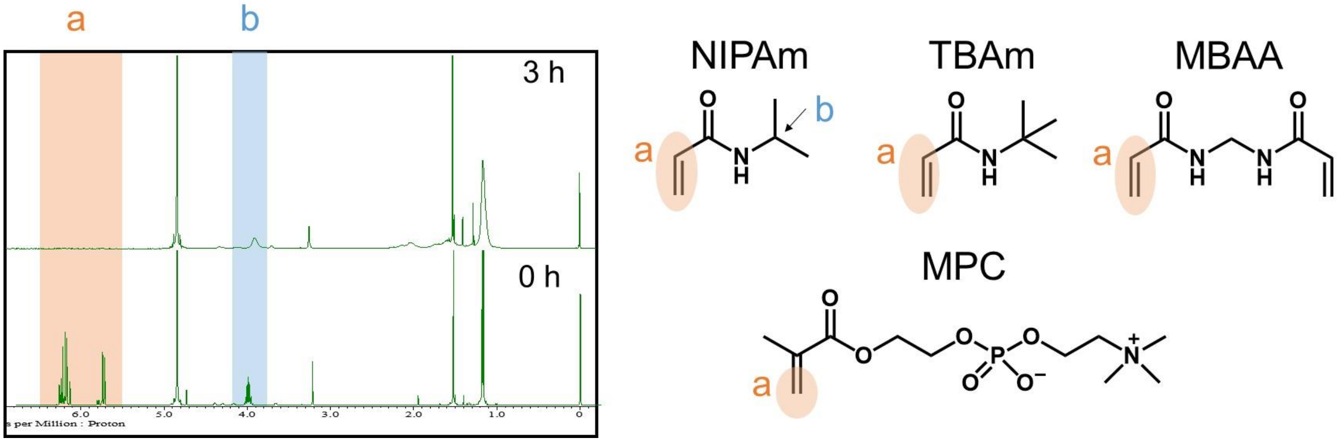

Figure S1 ^{1}H-NMR spectra of aqueous precipitation polymerization of water-soluble monomers (shown in this Figure) at different polymerization times (0, and 3 h).

2. ^{1}H-NMR in Dispersion Polymerization with PVA$_{1000}$

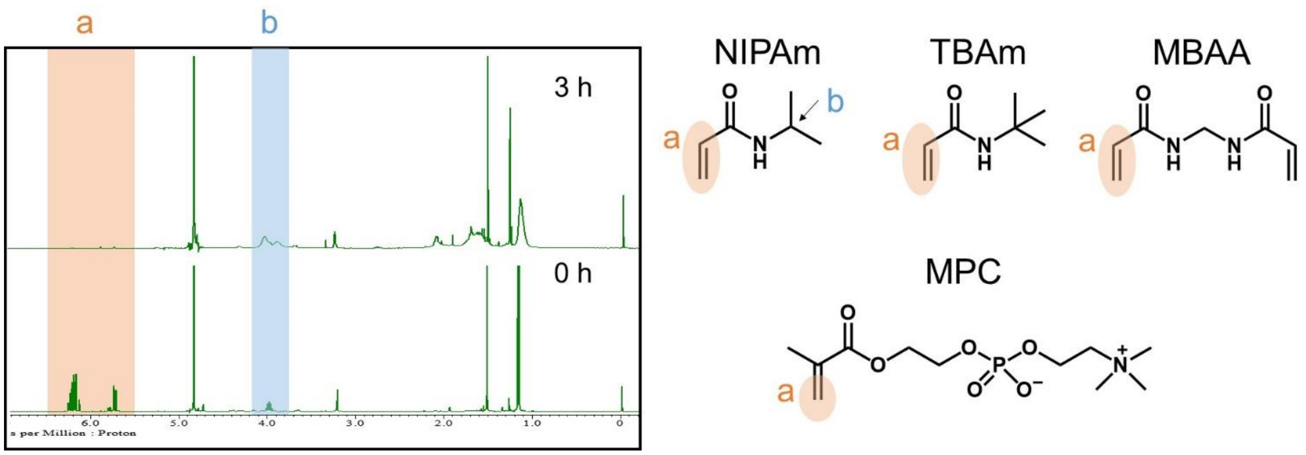

Figure S2 ^{1}H-NMR spectra of aqueous dispersion polymerization of water-soluble monomers (shown in this Figure) at different polymerization times (0 and 3 h) in the presence of PVA$_{1000}$.

3. Transmittance Measurements of PG_{1000}

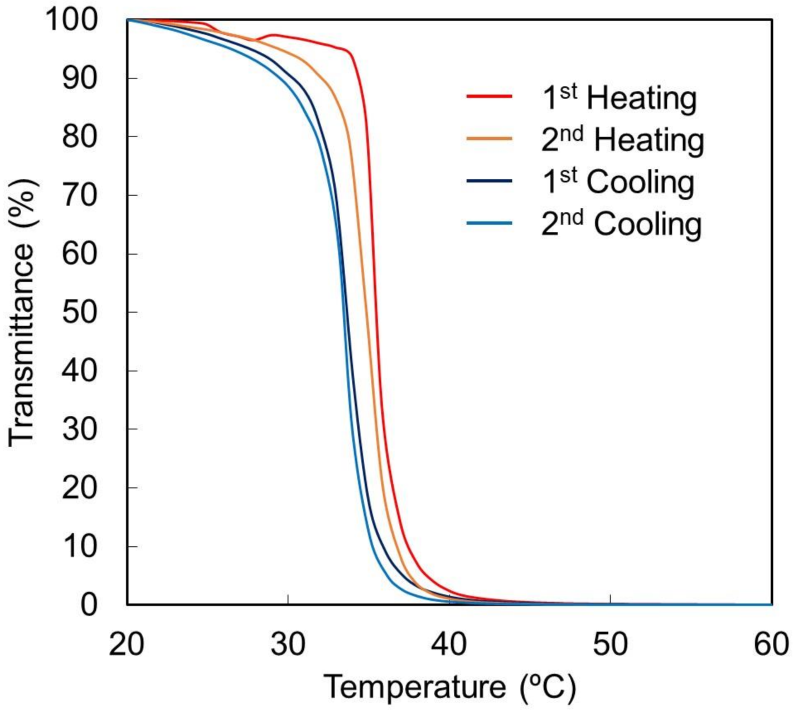

Figure S3. Transmittance change of polymer nanogels prepared by aqueous dispersion polymerization (with PVA_{1000}, 3mg/mL) at various temperatures. 1st heating (red), 1st cooling (navy blue), 2nd heating (orange), and 2nd cooling processes (blue).

4. Particle Size of PG_{1000} in the Presence of CTAB

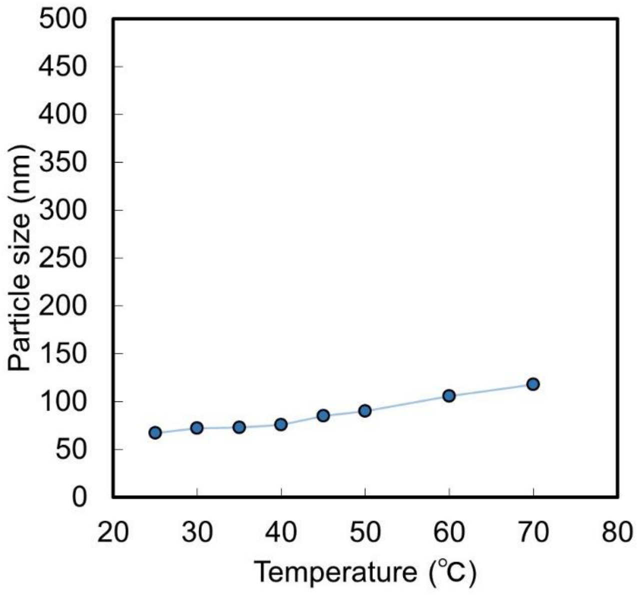

Figure S4. Averaged particle sizes of polymer gel particles at various temperatures in the presence of CTAB, where the polymer gel particles were prepared by dispersion polymerization with PVA_{1000} (3 mg/mL) at 70°C for 3 h.

5. Clouding-Point Temperature of PG_{1000} Prepared with Different PVA_{1000} Concentrations

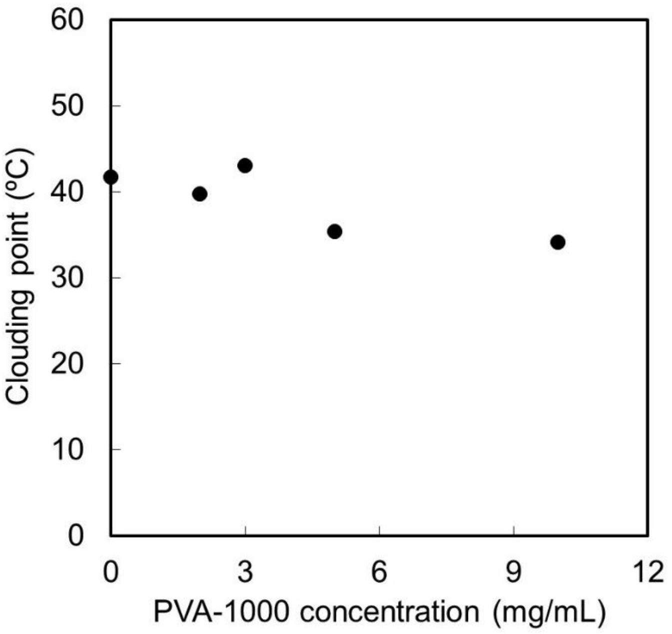

Figure S5. Clouding points of the dispersion containing polymer gel particles prepared by dispersion polymerization with different PVA_{1000} concentrations (2, 3, 5, and 10 mg/mL) at 70°C for 3 h.

6. Dispersion Polymerization with Fluorescein-labeled PVA_{500}

PVA_{500} (300 mg) was dissolved in DMSO (8 mL) at 95°C. Then, pyridine (50 µL, 62 µmol), dibutyltin dilaurate (1.9 µL, 3.2 µmol), and FITC (5.0 µL, 1.3 µmol) were added in the PVA_{500} solution. The reaction proceeded under 95°C for 2 h. After the reaction, 1-butanol was added to the solution to precipitate the polymer. The polymer was washed with 1-butal using centrifugation, and subsequent vacume-drying, resulting in the dry Fluorescein-labeled PVA_{500}. The dispersion polymerization was carried out using the Fluorescein-labeled PVA_{500} (3 mg/mL). NIPAm (407 mg, 3.6 mmol), TBAm (7.6 mg, 60 µmol), MPC (30 mg, 0.1 mmol), MBAA (30.8 mg. 0.2 mmol), and V-50 (217 mg, 0.8 mmol) were mixed with 10 mM phosphate buffer (pH 7.4, 100 mL) containig Fluorescein-labeled PVA_{500} (3 mg/mL) in a Schlenk flask. After N_2/vacuum cycles, polymerization was performed at 70 °C for 3 h.

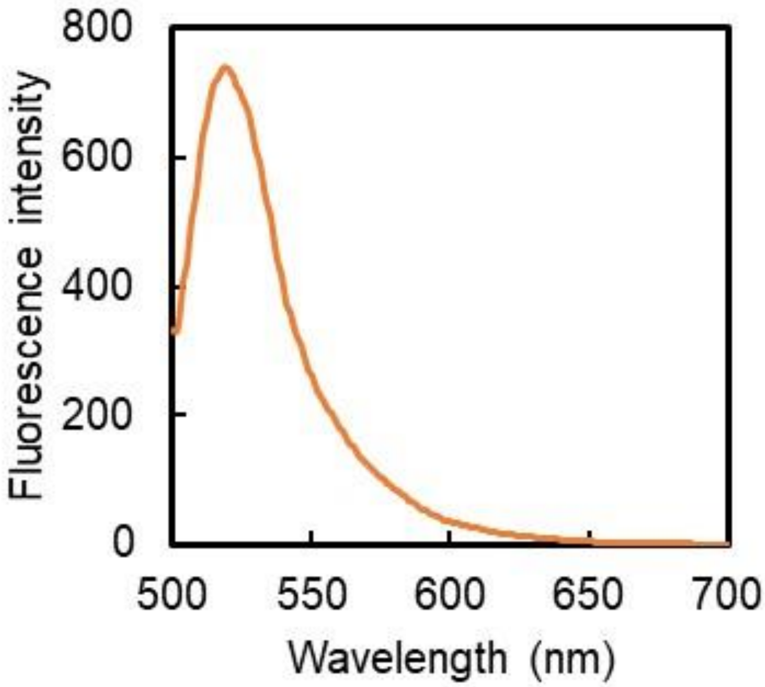

Figure S6. Fluorescence spectrum of polymer nanogels (PG_{500}) prepared by dispersion polymerization with FITC-PVA in 10 mM PB (pH 7.4) after removal of free FITC-PVA by ultracentrifugation.
